# Landscape Ecotoxicology of Coho Salmon Spawner Mortality in Urban Streams

**DOI:** 10.1371/journal.pone.0023424

**Published:** 2011-08-17

**Authors:** Blake E. Feist, Eric R. Buhle, Paul Arnold, Jay W. Davis, Nathaniel L. Scholz

**Affiliations:** 1 Northwest Fisheries Science Center, National Marine Fisheries Service, National Oceanic and Atmospheric Administration, Seattle, Washington, United States of America; 2 Washington Fish and Wildlife Office, United States Fish and Wildlife Service, Lacey, Washington, United States of America; Institute of Marine Research, Norway

## Abstract

In the Pacific Northwest of the United States, adult coho salmon (*Oncorhynchus kisutch*) returning from the ocean to spawn in urban basins of the Puget Sound region have been prematurely dying at high rates (up to 90% of the total runs) for more than a decade. The current weight of evidence indicates that coho deaths are caused by toxic chemical contaminants in land-based runoff to urban streams during the fall spawning season. Non-point source pollution in urban landscapes typically originates from discrete urban and residential land use activities. In the present study we conducted a series of spatial analyses to identify correlations between land use and land cover (roadways, impervious surfaces, forests, etc.) and the magnitude of coho mortality in six streams with different drainage basin characteristics. We found that spawner mortality was most closely and positively correlated with the relative proportion of local roads, impervious surfaces, and commercial property within a basin. These and other correlated variables were used to identify unmonitored basins in the greater Seattle metropolitan area where recurrent coho spawner die-offs may be likely. This predictive map indicates a substantial geographic area of vulnerability for the Puget Sound coho population segment, a species of concern under the U.S. Endangered Species Act. Our spatial risk representation has numerous applications for urban growth management, coho conservation, and basin restoration (e.g., avoiding the unintentional creation of ecological traps). Moreover, the approach and tools are transferable to areas supporting coho throughout western North America.

## Introduction

In recent decades, human population growth and development have continued to increase along the coastal margins of North America [1]. The associated changes in land cover and human land use have elevated land-based sources of pollution, and toxic stormwater runoff in particular, to become one of the most important threats to the biological integrity of basins, lakes, estuaries, and nearshore marine environments [2]. In the United States, concerns related to non-point source pollution have gained momentum over the past decade (e.g., [3], [4]). This has culminated most recently in the designation of “water quality and sustainable practices on land” as one of nine National Priority Objectives for the newly established National Ocean Council, together with ecosystem-based management, marine spatial planning, climate change and ocean acidification, and changing conditions in the Arctic [2]. For toxic runoff, however, the connections between unsustainable practices on land and the decline of ecological resilience in aquatic habits remain poorly understood.

In western North America, semelparous anadromous salmonids (*Oncorhynchus* spp.) typically migrate thousands of kilometers in their lifetimes. They hatch and rear in freshwater, migrate seaward to capitalize on the productivity of the oceans to grow rapidly and reach sexual maturity, and then return to their natal streams to spawn and die. Certain salmonids, including pink (*O. gorbuscha*) and chum (*O. keta*) migrate to the ocean relatively soon after hatching. Others, however, such as Chinook (*O. tshawytscha*), steelhead, (*O. mykiss*), sockeye (*O. nerka*), and coho (*O. kisutch*) may spend one or more years in freshwater lakes, rivers and streams. Because of this extended freshwater residency, juveniles of these species are potentially more vulnerable to anthropogenic modifications of freshwater habitat quality [5].

In contrast to the high mortality experienced by juvenile salmonids, mortality at the adult spawner life stage is relatively low. Familiar natural causes of mortality include predation, disease [6], [7], [8], [9], stranding (following high flows), elevated stream temperatures, and competition – e.g., in habitats with abundant salmon returns and limited spawning substrate. Various human activities such as recreational and commercial fishing, stream dewatering, and the placement of migration barriers can also increase salmon spawner mortality. In general, however, salmon spawner mortality has not been attributed to toxic chemical contaminants in stormwater runoff – a data gap that may be due, in part, to 1) the relative rarity of salmon spawners in urban basins with poor water quality, and 2) the logistical difficulty of implementing toxicity studies on migratory, seawater-to-freshwater transitional adults.

The exception is a recently documented phenomenon of returning adult coho salmon dying at high rates in urban and urbanizing streams in lowland Puget Sound region, which includes the greater Seattle metropolitan area [10]. Coho return to small coastal stream networks to spawn each fall. Entry into freshwater is triggered by early autumn rainfall and rising stream flows. Since there had been extensive habitat degradation and loss in these lowlands, many basins were targeted for stream restoration projects in the 1990s. Subsequent surveys to evaluate project effectiveness discovered that many coho salmon were dying in newly-accessible stream reaches before they were able to spawn – i.e., female carcasses were found in good condition (ocean bright colors) with skeins (membrane or sac that contains the eggs within the fish) filled with unspawned eggs [10]. In addition, affected coho from several different urban basins showed a similar progression of symptoms leading up to death, including disorientation, lethargy, loss of equilibrium, mouth gaping, and fin splaying. Systematic daily spawner surveys in recent years (2002–2009) have shown that adult mortality rates in urban streams are consistently high (relative to spawning coho salmon in more pristine areas), ranging from ∼25–90% of the total fall runs [10]. Mortality rates of this magnitude likely have important negative consequences for maintaining viable coho populations [11]. Consistent with this, most coho mortalities observed over the past decade were spawners that strayed (did not home to their natal stream reaches) into these restored urban freshwater habitats.

The precise underlying cause of recurrent coho die-offs remains under investigation. An initial weight-of-evidence forensic study has systematically ruled out stream temperature, dissolved oxygen, poor overall spawner condition, tissue pathology (e.g., gill), pathogen prevalence or disease, and other factors commonly associated with fish kills in freshwater habitats (Scholz et al., unpublished data). These findings, together with the rapid onset of the syndrome, the nature of the symptoms (e.g., gaping and disequilibrium), and the consistent re-occurrence within and between urban basins over many years together point to toxic stormwater runoff from urban landscapes as the likely cause of coho spawner mortality. Urban runoff and stormwater-influenced combined sewer overflows (CSOs) contain an exceptionally complex mixture of chemical contaminants. Specifically, urban streams are receiving waters for runoff and discharges containing pesticides [12], metals [13], petroleum hydrocarbons [14], plasticizers, flame-retardants, pharmaceuticals, and many other potentially toxic chemicals. The list of possible causal agents is therefore long.

The above chemical complexity notwithstanding, there are several reasons to suspect motor vehicles as sources of toxics that are killing returning coho. Vehicles deposit many compounds on road surfaces via exhaust emissions, leaking fluids, and the wearing of tires, brake pads and other friction materials [15]. Emissions contain nitrogen and sulfur dioxide, benzene, formaldehyde, and a large number of polycyclic aromatic hydrocarbons (PAHs). Fluids, including antifreeze and motor oil, contain ethylene and propylene glycol and PAHs. Tire wear releases zinc, lead, and PAHs onto road surfaces [16], and brake pad wear is a major source of copper, zinc, nickel, and chromium [16], [17]. Collectively, these contaminants accumulate on streets and other impervious surfaces until they are mobilized by rainfall and transported to aquatic habitats via runoff. Polycyclic aromatic hydrocarbons and metals such as copper are known to be toxic to fish, although acute lethality usually occurs at exposure concentrations that are higher (by orders of magnitude) than those typically detected in urban streams. It is likely that fall stormwater pulses contain higher concentrations than winter and spring due to the potential buildup of contaminants during the relatively dry summer months.

Although the adult die-off phenomenon has been observed in all Seattle-area urban streams where coho salmon occur, the overall rate of mortality has varied among basins. In qualitative terms, a higher proportion of returning animals have survived to spawn in basins that have more open space (e.g., parks and woodlands). Conversely, mortality rates have been consistently higher in basins with proportionately greater “urban” land cover and land uses. This raises the possibility of a quantitative relationship between discrete basin characteristics and coho survival and spawning success. Such a relationship would be important for several reasons. First, if coho mortality is significantly correlated with one or more land cover or land use variables, the latter could be used to identify unmonitored lowland basins where coho populations are at greatest risk. Second, it could provide a means to evaluate how future human population growth and development might impact wild coho populations in Puget Sound (and elsewhere) that are currently healthy. Finally, it could narrow the list of potentially causative pollution sources in urban basins, thereby focusing future toxicological studies to identify the specific contaminants involved.

In this study we performed a spatial analysis to identify landscape variables that correlate most closely with surveyed rates of coho spawner mortality across six different basins in Puget Sound. The variables included land use and land cover, tax parcel types, roadways, and impervious surfaces. We then used the information from these correlations to generate spatially explicit predictions of recurrent spawner losses in unmonitored basins throughout the four most densely populated counties in the greater Seattle metropolitan area.

## Materials and Methods

### Study Sites

**Figure 1:**
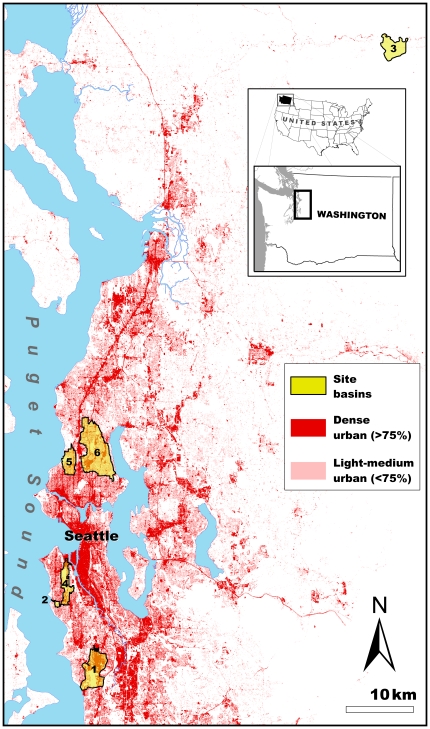
Six study sites where coho spawner mortality was monitored and landscape conditions were quantified. Main map depicts the Greater Seattle Metropolitan Area in Washington State, which is within the Puget Sound/Georgia Basin of the Pacific Northwest, United States of America (USA). Inset map illustrates location of the study sites within Washington State and the location of Washington State within the USA. For reference, red shading on main map represents the relative intensity of urbanization (light-medium and dense urban [23], [24]). Drainage basins depicted in yellow shaded polygons represent the total basin flowing into a given stream reach site. Key for site numbers: 1 = Des Moines; 2 = Fauntleroy; 3 = Fortson; 4 = Longfellow; 5 = Piper's; and, 6 = Thornton Creek.

We characterized habitat conditions within the drainage basins from streams at six sites in the Puget Sound lowlands (Figure 1). We chose these sites because coho spawner mortality has been monitored at these locations for several years (2000–2009; [10]). The sites represent a wide range of anthropogenic alteration, from highly urbanized (e.g., Longfellow Creek) to relatively undisturbed (e.g., Fortson Creek). Fortson Creek is considered a non-urban site, whereas the other five sites are urban streams and have varying degrees of development. The urban streams have all been a focus of varying restoration project efforts aimed at enhancing habitat quality for anadromous Pacific salmon. With the exception of the relatively unaltered Fortson Creek site, all site basins had impervious surface proportions well above the levels (5–10%) commonly associated with the decline of biological integrity in streams [18], [19].

Confirmed observation of the coho spawner mortality syndrome (see below) within a stream system was a key factor in study site selection. Importantly, natural production of coho in Seattle-area urban streams is very low. Not unexpectedly, recent modeling has shown that local coho population abundance declines precipitously at rates of spawner mortality documented for these drainages [11]. The adult returns to these streams are thus likely to be animals straying into sink or attractive nuisance habitats. Conversely, the syndrome has not been documented in streams where coho are relatively abundant – i.e., non-urban basins, as confirmed by a full season of daily stream surveys on Fortson Creek. Therefore, to evaluate the phenomenon in relation to land cover, we were constrained to streams where coho are affected, even if adult returns to these basins were low in certain years. Lastly, there is no evidence that the mortality syndrome is related to the origin of the spawners (i.e., hatchery vs. wild fish). For example, artificially propagated coho that return as adults to regional hatchery facilities in non-urban basins are unaffected.

### Study Subjects

Coho salmon in this study were all within the Puget Sound/Strait of Georgia Evolutionarily Significant Unit (ESU). An ESU is defined as a group of populations that 1) are substantially reproductively isolated from conspecific populations and 2) collectively represent an important component in the evolutionary legacy of the species [20]. Currently, Puget Sound/Strait of Georgia coho are designated a “species of concern” under the U.S. Endangered Species Act [21].

Coho typically spawn in small (lower order) streams in the Puget Sound lowlands in late fall and early winter and their fry emerge from stream substrates from March to May. Fry reside in riverine habitats for 14–18 months, smolt, migrate to marine environments where they grow rapidly and mature (16–20 months), and finally migrate to their natal basins where they spawn and die [22]. The adult spawners from the six study basins were both marked (adipose fin clipped) and unmarked, suggesting a mix of hatchery and wild origins.

### Coho Spawner Mortality

We used existing monitoring data collected as part of daily and weekly spawner surveys in each of the six study locations (Table 1). Data were collected during the fall spawning season from 2000–2009 by Seattle Public Utilities (SPU), the Wild Fish Conservancy, and the Northwest Fisheries Science Center (NWFSC). Streams were checked every few days in the early fall (usually the first or second week in October, depending on rainfall) until the first adult coho was observed. The streams were then surveyed daily for the duration of the fall run, until the last carcass was documented, typically in the first or second week of December. For several years, biologists working for the City of Seattle (Wild Fish Conservancy) also surveyed many of the same urban streams for coho spawner mortality on a weekly basis. Side-by-side comparisons of daily and weekly survey data (e.g., for Longfellow Creek in 2005 and 2007) revealed practically no loss of carcasses to scavengers. Accordingly, we included the weekly survey data in our analyses.

**Table 1:** Coho spawner mortality proportion and cumulative number of female carcasses enumerated (in parentheses) by site (columns) and year (rows).

	Des Moines	Fauntleroy	Fortson^1^	Longfellow	Piper's	Thornton
**2000**	-	0.25 (12)	-	0.74 (135)	0.18 (17)	0.88 (33)
**2001**	-	0.22 (9)	-	0.61 (111)	0.70 (37)	0.82 (11)
**2002**	-	0.00 (1)	0.01 (114)^a^	0.86 (57)^a^	0.60 (10)	080 (5)
**2003**	-	(0)	-	0.67 (18)^a^	0.00 (1)	1.00 (2)
**2004**	0.63 (30)^a^	(0)	-	0.89 (9)^a^	0.33 (3)	1.00 (1)
**2005**	-	0.75 (4)	-	0.72 (75)^a^	0.75 (4)	0.50 (8)
**2006**	-	(0)	-	1.00 (4)^a^	1.00 (9)^a^	1.00 (4)
**2007**	-	0.75 (4)	-	0.73 (41)^a^	0.20 (5)	0.80 (5)
**2008**	-	-	-	0.67 (12)^a^	-	1.00 (2)
**2009**	-	-	-	0.78 (36)^a^	-	-
**Overall**	0.63 (30)	0.37 (30)	0.01 (114)	0.72 (498)	0.57 (86)	0.83 (71)

A dash (-) indicates survey was not conducted for that year/site.

a Northwest Fisheries Science Center (NWFSC) daily surveys, all others were weekly and collected by Seattle Public Utilities (SPU) or the Wild Fish Conservancy [51], [52].

1 Non-urban site.

The entirety of the available spawning habitat within a given urban drainage was surveyed for premature adult coho mortality. For some streams, including Longfellow Creek, mid-stream barriers to upstream migration confined adults to the lower portions of the drainage. This made it possible, in the course of a few hours as part of a daily survey, to inspect all sections of the stream that 1) had a gravel substrate suitable for redds (spawning “nests” built by females), and 2) were focal areas for repeated (year-to-year) redd building during successive spawner runs.

Monitoring data were not collected at all sites for all years (Table 1). Mortality among returning coho was quantified only for females on the basis of egg retention – i.e., the number of partially spawned or unspawned female carcasses observed in streams over an entire spawning season. Notably, the total number of returning adults was low for some years and some basins (Table 1). Nevertheless, the aggregate spawner survey data used in this analysis are the most comprehensive currently available.

### Geospatial Datalayers

We used existing geospatial datalayers as our source of potential predictor variables and as a proxy for habitat type and condition. The datalayers were generated by a variety of organizations for planning and analytical purposes, making them suitable for running spatial analyses on habitat. They were also available over the entire spatial domain of our predictive model. We used four geospatial datalayers: Land-cover of the Greater Puget Sound Region [23], [24]; impervious and impacted surfaces [25]; property type (compiled from King [26], Kitsap [27], Pierce [28] and Snohomish county [29] tax parcel databases), and roadways (Puget Sound Regional Council; PSRC [30]).

The Land-cover of Puget Sound datalayer is the highest quality and most accurate depiction of land use and land cover in the Puget Sound lowlands. The datalayer used 30 m gridded LANDSAT TM imagery from 2002, which was extensively analyzed and corrected to produce an accurate (83% overall accuracy, [24]) depiction of land use and land cover conditions. To reduce the total number of potential predictor variables, we only used the dense urban (>75%); light to medium urban (<75%); and grass, crops and/or shrubs categories. We also combined the mixed and deciduous forest with the coniferous forest category and named it forests.

The impervious and impacted surfaces datalayer was derived from a 2001 LANDSAT TM image with 30 m pixels and an accuracy of 83–91% [25]. This datalayer depicts high to completely impermeable surfaces such as building roofs; concrete or asphalt roads and parking lots; concrete, asphalt or brick sidewalks, pedestrian walkways, and malls; etc.

One of the limitations of these two datalayers was that the pixel size of the source LANDSAT TM imagery is 30 m, so smaller features, such as roads and precise land cover boundaries, were not adequately captured. In order to address this deficiency, we analyzed property types and roadways, as they are represented as precise polyline and polygon delineations of the corresponding land cover variables. The boundaries in these geospatial datalayers were derived from precise survey data from major metropolitan areas, collected over many years by King, Kitsap, Pierce and Snohomish Counties.

The property types (parcels) datalayer was based on ground surveyed delineations of property, which are used for taxation purposes, with positional accuracy of +/−12 m or less [26], [27], [28], [29]. The original number of parcel types described by each county was between 103 and 292. Using the descriptions in the documentation that accompanied the datalayers, we were able to place each of the original parcel types into one of the five following categories: apartments and condominiums; commercial; industrial; parks and open space; and, residential.

The roadways datalayer was based on ground surveyed road and street centerlines. Each segment had a corresponding functional classification (FC##) code and width, as defined by the Federal Highway Administration [31] Highway Performance Monitoring System, and the Puget Sound Regional Council [30], respectively. We reduced the original nine functional classification types down to two categories: 1) heavily used roads (rural minor collector [FC08]; urban principal arterial - interstate [FC11]; urban principal arterial - other freeways and expressways [FC12]; urban principal arterial - other [FC14]; urban or rural minor arterial [FC16 or FC06]; urban collector [FC17]); and, 2) urban or rural local access roads (FC09 or FC19). We then calculated the total area (total length of given street centerline segment multiplied by its width) of each street functional classification for each corresponding site basin.

### Spatial Analyses

We defined the area of influence of the surrounding landscape for each site as the total area draining into that site (basin). Drainage basins for each site were generated using the ‘flowaccumulation’ command in Environmental Systems Research Institute (ESRI) ArcGIS (v. 9.3). We used a United States Geological Survey (USGS) 10 m digital elevation model (DEM) as the underlying terrain for generating basins. We then intersected the corresponding basin boundary for each of the six sites with each of the geospatial datalayers and their associated categories using ArcGIS. We quantified each geospatial datalayer and its associated category in a given basin as the fraction or proportion of the total area of the basin occupied by that geospatial datalayer or category. Longfellow Creek stood apart from the other sites in terms of the accuracy of the flow accumulation model because an unknown fraction of stormwater runoff in this drainage is diverted into the municipal sewer system. Therefore, the theoretical basin area, based on the terrain represented in the DEM, was not as representative of the true basin area compared with the other five sites.

### Statistical Analyses

We used generalized linear mixed-effects models (GLMMs; [32], [33]) to test the relationships between geospatial variables and coho spawner mortality. The response was binomial (observed number of female spawner mortalities each year, given the total number of female coho that returned to each site) and the models used a logit link function. All models included a random effect of site on the intercept, which accounts for nonindependence of the repeated samples taken at each site. We constructed a set of 139 candidate models by considering all combinations of the 12 predictors taken one, two, three or four at a time, with the restriction that a model could include at most one predictor from each of the four geospatial datalayers (land cover, impervious surfaces, property types, and roadways). We also excluded combinations of predictors that had a pairwise Spearman rank correlation exceeding 0.9 in absolute value. The candidate set included an intercept-only model as a no-effect baseline against which we could assess the predictive power of the geospatial variables.

We fitted the models using the Laplace approximation to the marginal likelihood [32] in the lme4 package in R [34], [35]. We then used Akaike's information criterion, corrected for sample size (AIC_c_) to rank the strength of evidence for each candidate model based on the data. Akaike's information criterion is a weight-of-evidence measure that reflects the balance between a model's goodness-of-fit to the data and its parsimony (i.e., number of parameters). Lower AIC_c_ values indicate greater support, and are reported as differences (ΔAIC_c_) relative to the best (smallest) value in the candidate set. We computed Akaike weights [36], which represent the relative support for each model, normalized so the weights sum to unity across the candidate set. We used these weights to compute model-averaged estimates and unconditional standard errors (SEs) for the fixed regression coefficients, and we quantified the relative importance of each predictor using variable weights (i.e., the summed Akaike weights of all models that included that predictor; [36]). These model averaging calculations were based on the 95% confidence set of models (i.e., the top-ranked models whose cumulative Akaike weight is 0.95), after re-normalizing the weights.

### Mapping coho spawner mortality

Using the fitted models, we built a map of predicted coho spawner mortality throughout the four counties (King, Kitsap, Pierce and Snohomish) representing much of the Puget Sound lowlands, by applying the GLMM equations to geospatial data from unmonitored basins. We used basins delineated in the National Hydrography Dataset Plus [37] as the underlying mapping unit (300 ha mean, 466 ha SD) and intersected the NHDPlus datalayer with each of the geospatial datalayers used in the statistical analyses. Within the four-county region, we only made spawner mortality predictions in basins where coho salmon presence has been documented, based on current geospatial datalayers generated by the Washington Department of Fish and Wildlife [38]. We then calculated the proportion of each basin that was covered by the selected landscape feature. We generated predicted values of the proportion of mortalities from each model in the 95% confidence set and then model-averaged these values using the normalized Akaike weights [36]. These predictions apply to the average basin in the Puget Sound coho ESU with some given set of habitat conditions, in the sense that the random effect of site was set to zero. To be conservative in representing the precision of the predicted values, we divided the calculated rates of likely coho spawner mortality into three bins: <10%, 10–50%, and >50%. These break points were chosen somewhat arbitrarily to represent low, medium and high spawner mortality rates.

## Results

**Figure 2:**
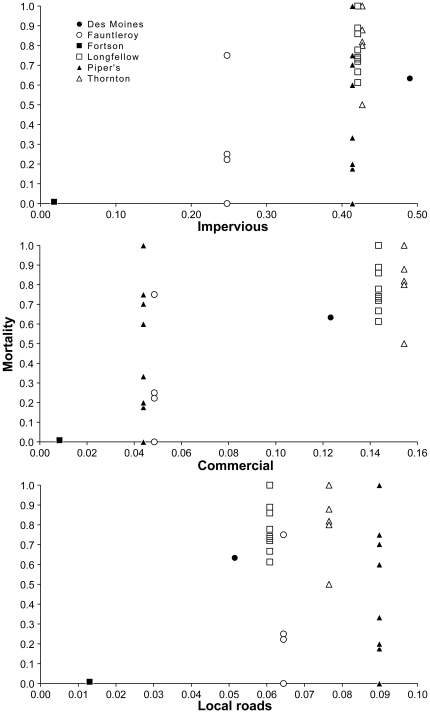
Female coho spawner mortality as a function of the proportion of each of the top three predictors in a given site basin, at the six study sites. Individual points correspond to specific years for each site. Mortality expressed as proportion of all returning females that died in a given year. Solid circle = Des Moines; hollow circle = Fauntleroy; solid square = Fortson; hollow square = Longfellow; solid triangle = Piper's; hollow triangle = Thornton Creek.

We found strong associations between land use and land cover attributes and rates of coho spawner mortality. Across the 95% confidence set of fitted models, three variables were particularly important for predicting mortality based on high variable weights: impervious surfaces, local roads, and commercial property type (Table 2 and Figure 2). There was substantial model selection uncertainty, reflected in a large 95% confidence set and large number of models with ΔAIC_c_<2.0 (37 and 8 of 139 candidate models, respectively; Table 3). In addition, although we excluded highly multicollinear combinations of variables (|*r*|>0.9), many variables were still strongly correlated, resulting in unstable parameter estimates and large unconditional SE estimates (Table 2). Nonetheless, predictive models that included land use and land cover attributes as predictors were clearly superior to the intercept-only model (ΔAIC_c_ = 20.4; Table 3), supporting the association of these variables with coho mortality.

**Table 2:** AIC weights, model averaged parameter estimates and unconditional confidence intervals for each variable, ranked by AIC_c_ weight.

			Model	
		AIC_c_	Averaged	Unconditional
Datalayer	Variable	weight	coefficient	SE
Impervious	Impervious surfaces	0.7158	16.8425	14.5376
Roadways	Local roads	0.5647	−15.6199	68.3331
Property type	Commercial	0.5107	7.9375	8.2616
Land cover	Dense urban	0.3865	−7.7776	16.1614
Property type	Apartments & condominiums	0.2409	−9.5330	31.1917
Roadways	Heavily used roads	0.2019	5.3445	31.5073
Land cover	Forest	0.1163	−0.7793	6.2249
Land cover	Light to medium urban	0.1149	0.3250	2.9751
Land cover	Grass, shrubs & crops	0.0993	0.1664	5.4517
Property type	Residential	0.0975	0.0738	16.8920
Property type	Industrial	0.0547	−0.2475	4.7008
Property type	Parks & open space	0.0000	0.0000	0.0000

**Table 3:** Summary of the 95% confidence set (37 of a total of 139 candidate models) of candidate models used to generate map of mortality rates, showing intercepts, estimated coefficients, ΔAIC_c_ and *w*_AICc_. Intercept only model included at bottom for reference.

Model	Equation	ΔAIC_c_	*w*_AICc_
a+b	−4.5664+19.76(a)+44.41(b)	0.000	0.0933
c+d+b	−3.9215−109.56(b)+48.75(c)−29.98(d)	0.046	0.0912
c+e+f	−3.9355+12.94(c)−40.15(e)+38.61(f)	0.372	0.0775
c+d+a	−4.4921+12.61(a)+14.03(c)−7.54(d)	0.579	0.0698
c+g+a	−4.4858+14.31(a)+5.23(c)+3.62(g)	0.669	0.0668
h+a+b	−2.6065+15.89(a)+30.87(b)−2.38(h)	1.150	0.0525
c+a+b	−4.6629+16.37(a)+35.26(b)+2.70(c)	1.357	0.0473
d+a+b	−4.7001+17.52(a)+43.83(b)+1.62(d)	1.576	0.0424
c+e	−4.5943+19.70(c)−53.28(e)	2.425	0.0277
c+d+i+b	−3.0628−83.44(b)+56.38(c)−40.28(d)−7.82(i)	2.485	0.0269
c+j+i+b	−7.3055−130.72(b)+21.23(c)+19.12(i)+10.65(j)	2.543	0.0262
c+d+k+b	−3.9266−94.52(b)+43.32(c)−25.00(d)−1.60(k)	2.613	0.0253
j+a+b	−4.5174+20.03(a)+43.79(b)−0.52(j)	2.752	0.0236
c+d+a+b	−4.0864+3.99(a)−76.44(b)+38.23(c)−23.27(d)	2.885	0.0221
c+d+a+f	−4.7368+15.57(a)+16.88(c)−9.22(d)−22.10(f)	2.925	0.0216
c+d+e+b	−3.9607−100.49(b)+46.40(c)−27.43(d)−5.54(e)	2.954	0.0213
c+d+e+f	−3.8347+12.37(c)+0.49(d)−40.69(e)+39.28(f)	3.280	0.0181
c+g+e+f	−3.8534+12.93(c)−40.45(e)+38.73(f)−0.18(g)	3.294	0.0180
c+j+e+f	−3.9360+12.94(c)−40.28(e)+39.36(f)−0.31(j)	3.326	0.0177
c+g+a+f	−4.6143+16.25(a)+5.79(c)−13.40(f)+4.06(g)	3.378	0.0172
c+d+i	−1.1996+64.26(c)−55.97(d)−24.83(i)	3.423	0.0168
h+i+b	9.3911−153.97(b)−17.49(h)+15.89(i)	3.858	0.0136
h+e+f	2.2747−27.99(e)+47.38(f)−7.31(h)	3.931	0.0131
h+a	1.2512+8.63(a)−6.13(h)	4.028	0.0124
c+j+a+b	−4.5887+16.71(a)+34.25(b)+2.72(c)−0.75(j)	4.299	0.0109
h+k+b	5.8364−27.35(b)−11.39(h)−5.97(k)	4.837	0.0083
c+j+e	−4.4356+18.70(c)−50.31(e)+1.33(j)	4.915	0.0080
c+j+k+b	−2.4511−52.30(b)+20.45(c)−13.34(j)−10.60(k)	4.937	0.0079
c+d+e	−4.7362+20.37(c)−0.45(d)−53.43(e)	5.141	0.0071
c+e+b	−4.4680−1.36(b)+19.52(c)−52.48(e)	5.158	0.0071
c+g+e	−4.5797+19.68(c)−53.23(e)−0.02(g)	5.188	0.0070
h+e+b	8.1285−20.52(b)−45.07(e)−14.67(h)	5.509	0.0059
c+k	−4.3426+13.30(c)−5.31(k)	5.649	0.0055
c+i+b	−5.6775−141.73(b)+22.77(c)+17.24(i)	5.821	0.0051
c+k+b	−3.9708−12.84(b)+14.63(c)−6.46(k)	5.896	0.0049
h+a+f	0.4930+6.87(a)+19.67(f)−5.22(h)	6.083	0.0045
c+d+i+f	−1.0499+68.65(c)−59.91(d)−6.04(f)−26.58(i)	6.343	0.0039
Intercept only	N/A	20.428	0

Model weights shown here are re-normalized for the set of 37 top-ranked models shown. a = commercial; b = local roads; c = impervious; d = dense urban; e = apartments and condominiums; f = heavily used roads; g = light to medium urban; h = forest; i = residential; j = grass, crops and/or shrubs; and, k = industrial.

**Figure 3:**
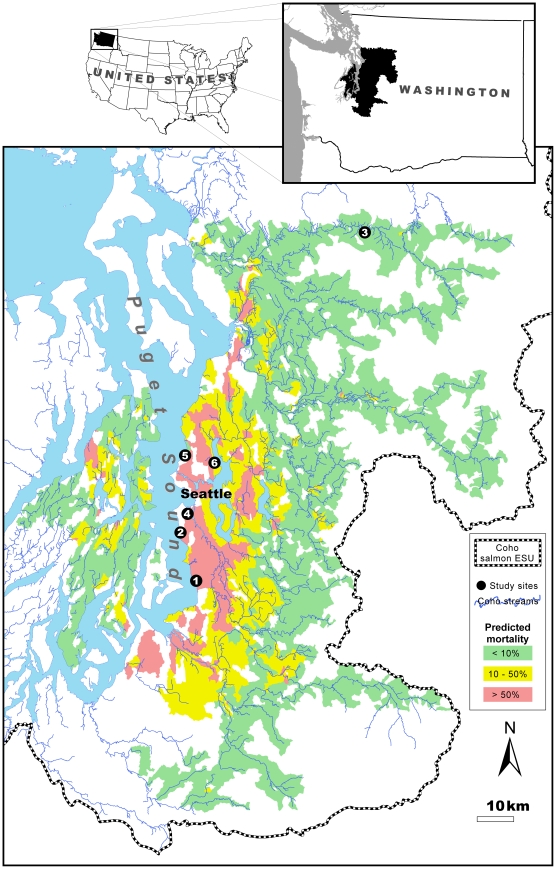
Predictive map of modeled coho spawner mortality rates within the Puget Sound lowlands. Mortality rates are a function of the proportion of key landscape variables within a given basin. Green, yellow and red areas indicate basins with predicted rates of spawner mortality (as a percentage of total fall runs) of <10%, 10–50%, and >50%, respectively. Black dots denote locations of the six study sites that were the basis for this analysis. Thick dashed black line depicts the southern boundary of the coho salmon Puget Sound/Georgia Basin Evolutionarily Significant Unit (ESU). Basins that do not have documented presence of coho salmon [38] are not represented on the map, even if they have landscape conditions associated with coho spawner mortality. Key for site numbers: 1 = Des Moines; 2 = Fauntleroy; 3 = Fortson; 4 = Longfellow; 5 = Piper's; and, 6 = Thornton Creek.

While the multicollinearity among potential predictors made causal interpretation of the models difficult, it did not preclude predictions of where coho salmon are likely to be affected along an urbanization gradient. Not surprisingly, the highest predicted mortality rates were clustered around the major metropolitan areas of eastern Puget Sound, contained within Snohomish, King, Kitsap, and Pierce counties (Figure 3). In addition, there is a significantly sized area in Eastern Puget Sound that has considerable proportions of the variables (local roads, impervious surface and commercial parcels) most correlated with substantial mortality rates. It is important to note that these predicted values have substantial associated uncertainty and should therefore be interpreted cautiously; however, it is reasonable to use them for assigning the break points for the low, medium, and high mortality rate categories represented on the map.

## Discussion

Overall, we have used conventional tools in landscape ecology to shed light on an unusually complex ecotoxicological challenge. Our analyses strongly suggest that specific characteristics of basins in the Puget Sound lowlands are linked to the die-offs of coho spawners that have been widely observed in recent years. Across basins, the strength of the association is greatest for impervious surfaces, local roads, and commercial property. We did not evaluate hydrologic or geomorphic basin characteristics as part of our analysis. Nevertheless, our findings support the hypothesis that coho are being killed by as-yet unidentified toxic chemical contaminants that originate from these types of surfaces and are transported to salmon spawning habitats via stormwater runoff.

Our results extend a large body of scientific information linking urbanization (broadly defined) and degraded water quality to a loss of biological integrity (sensu Karr [39]) and productivity in freshwater stream networks [18], [40], [41]. Previous studies have generally related land use and land cover variables to macroinvertebrate assemblages in streams [42], or to the relative abundance of salmon and other fish (e.g., [22], [43], [44]). The present analysis is novel because it relates basin characteristics directly to salmon health and survival, versus species presence or absence. Moreover, it offers new insights on the water quality aspects of urban runoff. The focus of most salmon restoration projects is physical characteristics of spawning and rearing habitat [45]. Most salmon specific restoration projects are deemed successful if they simply restore the physical habitat to a suitable state for a given species [46]. Our study suggests that suitable spawning and rearing habitat may not be supportive of coho salmon persistence when the surrounding landscape is urbanized. The linkages between increased impervious coverage within a basin, increased stormwater runoff, altered hydrologic processes, and ecological decline are well established (e.g., [18]). However, stormwater impacts encompass both physical and chemical drivers of decline, and it can be difficult to distinguish between these via *in situ* assessments because stream invertebrate communities integrate both stressor categories. Coho salmon spawners, by contrast, appear to be promising and specific sentinels for the degraded water quality aspect of urban runoff. Compared to macroinvertebrate sampling and taxa identification, the coho mortality syndrome is relatively easy and inexpensive for non-specialists to monitor in the form of digital video recordings of symptomatic fish, or the presence of unspawned female carcasses in streams.

Interestingly, the mortality syndrome appears to be specific to coho salmon. For example, there were temporally overlapping runs of coho and chum salmon (*O. keta*) in Piper's Creek in the fall of 2006. Whereas all of the adult coho succumbed to the mortality syndrome, the chum were unaffected, with nearly all surviving to spawn (130 of 135 spawned out female carcasses; Scholz et al., unpublished data). Consistent with this, the survey teams have not observed the characteristic symptoms (e.g., surface swimming, gaping) among other fish species that inhabit urban streams such as sticklebacks and cutthroat trout. Not only are coho unusual in this respect, the phenomenon appears to be restricted to the adult life stage. In the fall of 2003, surface flows from Longfellow Creek were diverted through streamside sheds housing aquaria that contained individual juvenile coho from the NWFSC hatchery. The juveniles (n = 20) were maintained and observed daily throughout the fall spawner run. Overall juvenile survival was 100%, and the juveniles behaved normally, even on days when symptomatic adults were observed in the nearby stream (Scholz et al., unpublished data). The underlying reasons for the syndrome's surprising uniqueness to adult coho are not yet known.

Daily or weekly stream surveys are labor intensive, and for this reason only a subset of urban drainages in Puget Sound have been monitored to date. The GIS-based mapping tool developed for this study can be used to focus future monitoring efforts on basins with a higher likelihood of coho die-offs based on land cover attributes. In addition to the basins we have identified within the range of the Puget Sound/Georgia Basin ESU, this approach could be extrapolated to other geographic areas where coho return to spawn along a gradient of urban growth and development. This includes, for example, coho from the Lower Columbia River ESU, a threatened population segment with a spawner range encompassing the greater metropolitan area of Portland, Oregon. Overall, future surveys will ground-truth initial model outputs and provide additional data that can be used to improve the predictive accuracy of the mapping tool.

Our findings have two near-term applications. First, they identify likely “hotspots” for coho spawner mortality throughout central Puget Sound. Given that recurring adult losses at a rate greater than approximately 10% are likely to substantially reduce local population abundances, the high mortality basins in Figure 3 (10–50% and >50% predicted mortality categories) may represent sink habitats for the Puget Sound/Georgia Basin ESU. This is an important consideration for coho recovery planning at the local, county, and regional scales. Second, our results indicate areas where toxic runoff could potentially undermine stream restoration efforts - specifically, strategies that improve physical and biological habitat conditions (flow, connectivity, channel complexity, riparian function, etc.) as a means to boost coho population productivity.

The potential influence of rainfall, including timing, frequency, and individual storm intensity, remains an area of active investigation. Throughout the years of stream surveys, it has been qualitatively evident that rainfall influences the mortality syndrome. For example, salmon that arrive and enter a stream during an extended dry interval (a week or more) often survive and then become symptomatic and die when it next rains (Scholz et al., unpublished data). One of our aims in surveying Longfellow Creek (the stream with the most abundant overall returns) for more than a decade was to evaluate inter-annual variation in coho spawner mortality in relation to rainfall. However, a quantitative analysis has proven problematic due to highly variable rainfall patterns in combination with low adult returns in some years. It is clear, however, that the syndrome is not a simple first-flush phenomenon. In most years, both egg retaining and spawned out carcasses were observed across the 8–10 week fall run, irrespective of the number and size of rain events over that interval.

Over the longer term, an approach similar to the one developed here could be used to forecast the likely impacts of future human population growth and development on Puget Sound coho populations that are currently healthy. For example, the expansion of local road networks is a core focus for urban growth planning, and these projections could serve as a basis for evaluating how and where coho spawner mortality will increase under different growth management scenarios. This, in turn, would inform strategies to reduce or mitigate toxic runoff in highly productive basins, in advance of expanding transportation infrastructure – i.e., prevention vs. costly retrofits to the built environment. Also, our modeling approach could be expanded to include the timing and intensity of rainfall as potential drivers for coho spawner mortality. Rainfall patterns may be a key determinant of stormwater quality, although more work in this area is needed. Climate change is expected to shift regional rainfall patterns, and it should be possible to explore how this will interact with changing land cover (urbanization) to influence stormwater quality and toxic runoff to coho spawning habitats.

While not definitive, our results reinforce the parsimonious explanation that coho deaths are caused by one or more contaminants originating from motor vehicles. As noted earlier, this is important because it narrows the list of candidate toxics in complex urban landscapes. Future toxicological studies should focus on two ubiquitous urban runoff contaminant classes in particular. The first are metals in brake pads and other vehicle friction materials. Copper, zinc, and other metals are known to specifically target the fish gill, thereby disrupting respiration and osmoregulation [47]. The second, PAHs, [14], [48], [49] are taken up across the fish gill, and can impair cardiac function and respiration [50]. The symptoms displayed by affected coho (surface swimming, gaping, loss of equilibrium, etc.) are consistent with a disruption of respiration, osmoregulation, or circulation, or some combination of these.

Notably, PAHs and metals usually cause the above toxicological effects at concentrations well above those typically detected in urban streams. However, the majority of conventional toxicology studies using salmonids focus on freshwater species (e.g., rainbow trout) or the freshwater life stages of juvenile anadromous species. There are practically no toxicity data for coho salmon at the adult spawner stage. Many important osmoregulatory changes take place during the transition from seawater prior to spawning, and these may render adult coho more vulnerable to metals and PAHs than freshwater-resident salmonids. Adding to this complexity is the possibility of interactive toxicity (e.g., synergism) among contaminant mixtures. Studies that experimentally reproduce the familiar symptomology and mortality in adult coho, under controlled exposure conditions with environmentally realistic mixtures of metals and PAHs, will likely be necessary to definitively implicate motor vehicles.
